# The Impact of Social Support: Fathers’ Depressive Symptoms and Parenting Stress

**DOI:** 10.1177/0192513X251322143

**Published:** 2025-03-18

**Authors:** Emily Hogan, Dana Ronaghan, Karis Cochrane, Alyssa Romaniuk, Lara Penner-Goeke, Taryn Gaulke, Jennifer Theule

**Affiliations:** 1 Department of Psychology, 8664 University of Manitoba, Winnipeg, Canada

**Keywords:** parenting stress, fathers, social support, depressive symptoms

## Abstract

Parental depressive symptoms are positively correlated with levels of parenting stress. Greater parenting stress predicts negative outcomes for both parents and children, and worse family functioning. Social support is a key protective factor against depressive symptoms; however, minimal research has examined the relationship between social support, paternal depressive symptoms, and parenting stress among fathers. Seventy-nine fathers of children, aged 2–6 years old, completed an online survey. Mediation analyses, using Hayes’ PROCESS macro, determined significant indirect effects of social support on parenting stress through paternal depressive symptoms. Our findings elucidate the need for social support from family, friends, and significant others to improve depressive symptomology and parenting stress among fathers. We encourage fathers to seek support from friends, family, and their partners, to benefit their mental health and the family unit. Clinicians working with fathers should be aware of the possibility of co-occurring problems related to these factors.

## Introduction

Since the start of the COVID-19 pandemic, the percentage of individuals experiencing a major depressive disorder, and the average level of depressive symptom severity in the general population, have increased (Ettman et al., 2020; Santomauro et al., 2021). The 12-month prevalence of major depression is similar across Canada (8.2%) and the United States (8.7%; Vasiliadis et al., 2007). While rates of depression are lower in men compared to women, rates of suicide are three times higher among men (Albert, 2015; Government of Canada, 2020). Men exhibit different patterns of depressive symptom prevalence and severity (Martin et al., 2013) and are less likely to seek help for their symptoms than women (Call & Shafer, 2015); however, men’s mental health remains understudied (Smith et al., 2018). Additionally, the mental health of fathers is of significance, as men at the greatest risk for depression are those within the age group most likely to have children (i.e., men younger than 45-year old; Kane & Garber, 2004). Fathers are an important part of a family system with research showing that children who have involved and engaged fathers exhibit more positive physical, cognitive, and emotional outcomes than those who do not (Campbell et al., 2015). Conversely, fathers’ poor mental health can contribute to more internalizing and externalizing behaviors in children (Fisher, 2017).

Social support is a key protective factor against depressive symptoms and clinically significant depression (Schiller et al., 2021). Social support refers to the perceived or received psychological and material resources available through one’s family, friends, and partner (Dunne et al., 2018). It can help individuals feel connected and appreciated and is correlated with positive mental health and quality of life (Alsubaie et al., 2019). It is essential for increasing families’ emotional and material resources and connectedness (Tracy, 1990). Greater levels of perceived social support are correlated with lower depressive symptom severity and duration of symptoms, improved functioning, and depressive disorder recovery (Fowler et al., 2013; Wang et al., 2018). Specifically, among men diagnosed with depression, social support from friends and family has been found to be integral in decreasing depressive symptoms and coping with daily life (Skärsäter et al., 2003). However, compared to women, men often have smaller, less supportive social networks and fathers may perceive less social support and more social isolation than mothers (Fierloos et al., 2022). Depressed parents experience high levels of fatigue, loss of interest, and negative mood, which can lead to decreased engagement with their children (Nelson et al., 2009). Parental depressive symptoms affect the individual experiencing them and become a source of stress for the family unit (Nelson et al., 2009). Indeed, families including a member with a depression diagnosis report worse family functioning compared to families without a depression diagnosis (Downey & Coyne, 1990). Further, parental depressive symptoms have been found to predict parenting stress (PS; Yim et al., 2015).

PS describes a parent’s aversive psychological response to the overwhelming demands of parenthood (Deater-Deckard, 1998). It is a continuous variable, affecting all parents to some degree and is distinct from other areas of stress and family functioning (Deater-Deckard, 1998). PS is influenced by various interrelated parenting experiences, including parenting task demands, the psychological well-being of both the parent and child, and the quality of the parent-child relationship (Deater-Deckard, 1998). There is a positive association between parental depressive symptoms and levels of PS (Lim & Shim, 2021). Greater PS predicts less warm parenting behaviors, negative outcomes for both parents and children, and worse family functioning (Harewood et al., 2017; Lim & Shim, 2021; Stone et al., 2016). Further, PS reduces parenting and life satisfaction (Crnic & Greenberg, 1990). Despite the existing literature, there remains little known about the impact of fathers’ depressive symptoms on the relationship between social support and PS.

### Depressive Symptoms

According to the Diagnostic and Statistical Manual of Mental Disorders (DSM-5-TR), depressive symptoms include depressed mood, diminished pleasure in activities, insomnia, difficulty concentrating, and feelings of worthlessness (American Psychiatric Association, 2022). Fathers who live with their children have higher depressive symptom scores than nonfathers (Garfield et al., 2014). Of note, depressive symptoms exist on a continuum; that is, individuals can experience symptoms without meeting diagnostic criteria, but still suffer from significant functional impairment and psychosocial dysfunction (Rodriguez et al., 2012).

### Social Support and Depressive Symptoms

Many fathers lack social support and connections from whom they can seek encouragement or emotional support (Levy & Kotelchuck, 2022). Among fathers, limited social support and a small social network are associated with higher levels of depressive symptoms (Kim & Swain, 2007). The source of support plays a crucial role in determining the perceived quality of social support (Tracy, 1990). Men perceive and seek social support from different sources than women; however, the key sources men seek support from differ between studies (Alsubaie et al., 2019).

A qualitative study found that depressed fathers prefer to seek support from informal supports such as significant others, family, or friends over formal support and professional services (Rominov et al., 2018). More specifically, a Dutch study found that men receive most of their support from their spouses while women receive most of their support from relatives and friends (Van Daalen et al., 2005). Conversely, Alsubaie et al. (2019) found that among individuals attending university, men perceived significantly less social support from their significant others compared to women. Mixed results exist on the sources from which fathers seek social support (Darwin et al., 2017; Rominov et al., 2018).

Sources of social support have differential effects on well-being and quality of life across various populations (Leung et al., 2020; Yuh & Choi, 2017). Further illustrating the unique experiences of fathers, depressed fathers are often discontent with their perceived social support and their depressed mood is associated with a discrepancy between their social life expectations around the birth of their first child and their actual experience (Bielawska-Batorowicz & Kossakowaska-Petrycka, 2006). Further, Castle et al. (2008) found that while perceived social support is a protective factor against depressive symptoms for fathers, their perceived emotional social support decreases during and after their partners’ pregnancy; this study suggests this is because fathers’ primary supporter, their partner, become less available to provide them emotional support when their partner’s attention is focused on their child’s needs.

Some fathers cope with stress through distraction or denial instead of seeking parenting support (Darwin et al., 2017). When men become fathers, they are often only recognized as support providers for their partners (Kim & Swain, 2007). This lack of social support and the expectation to fulfill a supporter role may be detrimental as fathers are vulnerable to postpartum depression when they experience excessive stress, feel excluded from the mother–child relationship, and have low self-esteem in their role as a father (Kim & Swain, 2007; Wang & Chen, 2006).

### Depressive Symptoms and Parenting Stress

PS arises as parenting demands (i.e., a child’s need for survival and affection) exceed the available resources needed to meet them (i.e., sufficient housing, knowledge, and tangible or emotional social support; Deater-Deckard, 2004). However, not all parents perceive daily stressors to the same extent. Specifically, parents with depressive symptoms are more likely to experience heightened PS (Crnic & Ross, 2017; Shea & Coyne, 2011; Yim et al., 2015). Symptoms of depression, such as negative biases and low self-esteem, may exacerbate fathers’ experiences of PS (Deater-Deckard, 2004). Greater depressive symptoms are linked to decreased parenting self-efficacy, strained marital relationships, and overall, more stressful life experiences (Choi & Marks, 2008; Crnic & Ross, 2017; You & Conner, 2009), which contribute to overwhelming PS (Crnic & Ross, 2017; Deater-Deckard, 2004). Among parents diagnosed with depression, greater symptom severity is related to decreased psychological resilience and family functioning (Jaser et al., 2005), impacting their ability to recover from stress both as an individual and as a family unit. Compared to mothers, fathers may also face additional challenges coping due to gendered expectations; for example, fathers may question whether their PS is legitimate, face stigma around depression and stress, and may feel pressure to ‘man up’ and ignore their emotions (Darwin et al., 2017). Paternal PS also predicts fewer hours of fathers’ available time to spend with their children (Halme et al., 2006). In addition to finding parenting to be more stressful, fathers with depression also experience greater aggravation related to parenting than fathers without depression (Bronte-Tinkew et al., 2007). Fathers’ depressive symptoms are correlated with stress related to the role of parenthood (Biondic et al., 2019).

### Social Support and Parenting Stress

A negative relationship exists between social support and PS (Solmeyer & Feinberg, 2011; Song et al., 2021). When parents feel supported, they are better equipped to manage child-rearing difficulties and they experience stronger parenting self-efficacy, which is also correlated with lower PS (Fang et al., 2021; Solmeyer & Feinberg, 2011). Despite the existing literature on the associations between social support and PS, it is worth exploring the role of depressive symptoms in this relationship, as depressive symptoms also contribute to the hypothesized mechanisms for this relationship (i.e., self-efficacy; O’Neil et al., 2009). Generally, depressive symptoms can impact one’s perception and memory, resulting in a preference for negative rather than positive information (Gotlib & Joormann, 2010). Therefore, it is important to consider the role of depressive symptoms in the relationships between social support and PS as they may potentially influence this association.

### Current Study

Research has identified an association between social support and paternal depressive symptoms (Castle et al., 2008), as well as between paternal depressive symptoms and PS (Crnic & Ross, 2017). However, there is a significant gap in the existing literature which motivated our study to analyze the mediating effect of paternal depressive symptoms. Specifically, our study looked at these relationships with social support from several sources: friends, family, and significant other. We aimed to develop a broader understanding of family systems from a paternal mental health perspective. By focusing on fathers of typically developing children from two to six years old, this study aimed to expand understanding of the role of paternal depressive symptoms in family systems, as most previous literature focuses on fathers of newborns and toddlers. This study focused on fathers of children in the preschool developmental stage, as this is a stage in family development with significant changes (Duvall, 1988). Moreover, this study utilized a symptomatically diverse group of fathers, investigating the role of paternal depressive symptoms across the spectrum from general wellness to clinically significant symptoms.

To reach our objectives, the following questions were addressed: (1) Are social support (total, and from friends, family, or significant other) and paternal depressive symptoms correlated? (2) Is social support from any source and PS correlated? (3) Are paternal depressive symptoms and PS correlated? (4) Is the association between social support from any source and PS mediated by depressive symptoms? Given the preceding literature, it was predicted that: (1) Social support from each source and depressive symptoms would be negatively correlated. (2) Social support from each source and PS would be negatively correlated. (3) Paternal depressive symptoms and PS would be positively correlated. (4) Social support from each source would have an indirect effect on PS through paternal depressive symptoms.

## Method

### Sample

The sample included 79 fathers residing in Canada or the United States with a typically developing child between the ages of two and six years old. This study was part of a larger study which collected data from a variety of populations and used measures that were not included in this study. Fathers were recruited online and in-person via national and provincial parenting organizations, schools, and social media. Exclusion criteria included fathers who are younger than 18 years old, have custody of their child less than 50% of the time, have had custody for less than four months (Vanschoolandt et al., 2013), live with their co-parent less than 50% of the time, have a child with a diagnosis of autism spectrum disorder (ASD) or attention deficit hyperactivity disorder (ADHD), or have a child who met cut off criteria for measures of ASD (*Lifetime Social Communication Questionnaire*; Rutter et al., 2003), ADHD (*ADHD Rating Scale-IV: Home Version*; DuPaul et al., 1998a), or intellectual developmental disorder (*The Developmental Profile, Fourth Edition*; Alpern, 2020).

### Procedure

The preceding research questions were addressed through a cross-sectional survey. Participants followed instructions on a recruitment poster to complete a series of demographic questions and measures. Upon agreeing to complete the survey, participants were able to enter a draw to win one of ten gift cards (US$50) through their email. This study was reviewed and approved by the University of Manitoba’s Psychology/Sociology Research Ethics Board.

### Measures

#### Demographics Questionnaire

A demographics questionnaire was used to collect self-report data from fathers on the following variables: age, ethnicity, child gender, child diagnoses, and family composition.

#### ADHD Rating Scale-IV: Home Version

The ADHD Rating Scale-IV: Home Version (DuPaul et al., 1998a) assessed overall level of ADHD symptomology in participants’ children. This was used to screen out fathers of children with clinically significant symptoms of ADHD. The 98^th^ percentile was used as a cutoff point for ADHD (DuPaul et al., 1998a). This scale has adequate internal consistency (α = .92).

#### The Lifetime Social Communication Questionnaire (SCQ)

The SCQ assessed symptoms of autism spectrum disorder (Rutter et al., 2003). This was used to screen out fathers of children with clinically significant symptoms of ASD. A total score of 15 was used as a cutoff point for ASD in the present study (Hollocks et al., 2019). The SCQ discriminates well between ASD and non-ASD cases and has a sensitivity of .88 and specificity of .72 (Chandler et al., 2007).

#### The Developmental Profile, Fourth Edition (DP-4)

The DP-4 assesses five domains of child functioning (Alpern, 2020). The present study only used the cognitive domain parent/caregiver checklist. Fathers who reported a score below 85 for their child were excluded. Internal consistency of the cognitive parent/caregiver checklist is strong (α = .82–.95; Western Psychological Services, 2020).

#### Center for Epidemiologic Studies Depression Scale (CES-D)

The CES-D measured participants’ self-reported depressive symptoms (Radloff, 1977). This scale includes 20 items on experiences with symptoms of depression within the past week on a four-point scale. Scores on the CES-D are continuous and range from 0–60. Higher scores indicate greater depressive symptoms. The CES-D has high internal consistency in the general population (α = .85), moderate test-retest validity (α = .45–.70), good concurrent validity, and adequate construct validity (Radloff, 1977).

#### The Multidimensional Scale of Perceived Social Support (MSPSS)

The MSPSS assessed subjective ratings of the adequacy of perceived social support (Zimet et al., 1988). This is a self-report scale which measures three subgroups of perceived support from family, friends, and a significant other. Each subscale consists of four distinct items. The present study used the total MSPSS scale and the three subscales. The total MSPSS scale includes 12 items which are rated on a 7-point Likert scale. Total scores range from 12 to 84, with higher scores indicating higher levels of perceived social support. The MSPSS total scale and subscales have high internal consistency (α = .85–.91), test-retest reliability (α = .72–.85), and adequate construct validity (Zimet et al., 1988).

#### Parenting Stress Index 4 – Short Form (PSI-4-SF)

The PSI-4-SF measured parent-reported perceived stress around parenting (Abidin, 2012). It was developed for parents of children from birth to 12 years old. It consists of 36 items on three domains including parental distress, parent–child dysfunctional interaction, and difficult child. For 33 items, participants report their experienced stress on a 5-point Likert scale. For three items, parents report experiences with stress on a five-point scale each with unique question-specific descriptions. Scores range from 36 to 180 and higher scores indicate greater PS. The total PSI-4 has excellent internal consistency (α = .96) and test-retest reliability (α = .65–.96; Abidin, 2012).

### Data Analysis

We used Statistical Package for the Social Sciences (SPSS) to analyze data. Mediation model analyses using PROCESS macro model 4 (Hayes, 2017) were used to determine the indirect, direct, and total effects of social support on PS while paternal depressive symptoms served as the mediator variable. This moderated mediation was conducted four times, each time with social support from a different source.

### Transparency and Openness

We report all measures in the study, and we follow Journal Article Reporting Standards (Applebaum et al., 2018). Study materials are available from cited sources; our study code and data are not publicly available. This study was not pre-registered.

## Results

### Descriptive Statistics and Direct Effects

Fathers were predominantly White (73.4%; *n* = 58), on average 34.38 (*SD* = 6.53) years old, were partnered (89.8%; *n* = 71), had a co-parent (96.2%; *n* = 76), had completed post-secondary education (77.2%; *n* = 61), and made above US$90,000 annual income (72.2%; *n* = 57). On average, fathers had 1.80 (*SD* = 0.74) children. Parents completed questionnaires with a target child in mind. Target children averaged 3.42 (*SD* = 1.35) years old. Table 1 provides descriptive statistics.

**Table 1.** Participant Demographics.

Variable	*n*	%
Gender of selected child		
Boy	41	51.9
Girl	38	48.1
Ethnicity		
Asian	11	13.9
Black	2	2.5
White	58	73.4
Multiracial	4	5.1
Other	4	5.1
Gender of co-parent		
Man	2	2.9
Woman	66	97.1
Receiving parenting services		
Seeking formal parenting support	29	36.7
Belong to a social media parenting group	31	39.2
Belong to an online or in-person parenting group	27	34.2
Seeing a psychologist, therapist or parent educator for parenting-related concerns	16	20.3
At least one of the parenting services listed above	47	59.5
None of the parenting services listed above	32	40.5
Recruitment source		
Social media (Facebook, Twitter, Instagram)	66	83.5
Webpage posting	2	2.5
Community posting (school, daycare, and community center)	4	5.1
Other or prefer not to answer	7	8.9

Fathers’ average social support – total fell within a high range (MSPSS >5.1; Fierloos et al., 2022). They reported significantly more social support – significant other compared to social support – friends (*t(77)* = −3.17, *p* = .002), and compared to social support – family (*t(77)* = −2.19, *p* = .032), as shown by paired samples *t*-tests. While fathers reported more social support – family than social support – friends, this difference was non-significant (*t(77)* = −1.65, *p* = .104).

Fathers ranged in depressive symptoms from zero reported symptoms to symptoms that align with clinically significant major depression in previous literature (CES-D >20; Vilagut et al., 2016). Please see Table 2 for variable descriptives.

**Table 2.** Descriptive Statistics for Major Study Variables.

Variable	Range	Mean	*SD*
Depressive symptoms (CES-D)	0 to 44 (scale ranges from 0–60)	16.31	12.27
Social support–Total (MSPSS)	28–84	65.19	14.72
Social support–Family (MSPSS)	2.25 to 7	5.44	1.37
Social support–Friends (MSPSS)	2 to 7	5.22	1.46
Social support–Significant other (MSPSS)	2 to 7	5.64	1.29
Parenting stress (PSI-4-SF)	36 to 142 (scale ranges from 36–180)	86.20	25.16

*Note.* CES-D = Center for Epidemiologic Studies Depression Scale, MSPSS = Multidimensional Scale of Perceived Social Support, PSI-4-SF = Parenting Stress Index Fourth Edition Short Form.

A correlation matrix was calculated to determine the relationship between social support total and from each of family, friends, and significant other; paternal depressive symptoms; and PS. Please see Table 3 for correlations. While some correlations were non-significant, Hayes (2017) states that correlation between variables is no longer considered a required precondition for conducting mediation analyses. Given that all correlation coefficients were below .800 (Table 3), multicollinearity did not exist between major variables (Senaviratna & A Cooray, 2019).

**Table 3.** Correlation Matrix of Variables.

Variable	1	2	3	4	5	6	7	8	9
1. Paternal depressive symptoms	—								
2. Social support–Total	−.226*	—							
3. Social support–Family	−.197	^a^	—						
4. Social support–Friends	−.192	^a^	^a^	—					
5. Social support–Significant other	−.219	^a^	^a^	^a^	—				
6. Parenting stress	.650**	−.399**	−.399**	−.278*	−.386**	—			
7. Parenting stress–Parent distress	.738**	−.452**	−.409**	−.399**	−.388**	^a^	—		
8. Parenting stress–Parentchild dysfunctional interaction	.510**	−.275*	−.315**	−.113	−.315**	^a^	^a^	—	
9. Parenting stress–Difficult child	.599**	−.069	−.093	−.003	−.095	^a^	^a^	^a^	—

^a^These correlations came from the same questionnaire and were therefore not included.

*p < .05, **p < .01.

### Mediation Analysis (Conditional Process Analysis)

Mediation analyses using Hayes (2017) PROCESS Model 4 were conducted to determine whether there is an indirect effect (mechanism pathway) through which social support affects PS via paternal depressive symptoms. Five thousand bias-corrected bootstrap samples were requested.

#### Analyses Model 4.1 – Social Support – Total and Parenting Stress, Mediated by Paternal Depressive Symptoms

The indirect effect of social support – total on PS through paternal depressive symptoms was significant, as demonstrated by the confidence index not straddling zero; *b* = −.487, *SE* = .141, 95% CI [−.7926, −.2379], (*n* = 70). The conditional direct effect of social support – total on PS (c’ path) was non-significant *b* = −.183, *SE* = .181, *t(67)* = −1.01, *p* = .316. Upon examining the individual pathways for this model, the pathway between social support – total and paternal depressive symptoms (a path) was significant (*b* = −.356, *SE* = .078, *t(68)* = −4.56, *p <* .001), and the pathway between paternal depressive symptoms and PS (b path) was significant (*b* = 1.368, *SE* = .246, *t(67)* = 5.567, *p <* .001). These results indicate that the relationship between social support – total and PS is fully mediated by paternal depressive symptoms. Please refer to supplemental materials for more information.

#### Analyses Model 4.2 – Social Support – Family and Parenting Stress, Mediated by Paternal Depressive Symptoms

The indirect effect of social support – family on PS through paternal depressive symptoms was significant; *b* = −4.658, *SE* = 1.528, 95% CI [−7.973, −2.079], (*n* = 70). The conditional direct effect of social support – family on PS (c’ path) was non-significant *b* = −2.491, *SE* = 1.878, *t(67)* = −1.327, *p* = .189. The pathway between social support – family and paternal depressive symptoms (a path) was significant (*b* = −3.448, *SE* = .862, *t(68)* = −4.003, *p <* .001), and the pathway between paternal depressive symptoms and PS (b path) was significant (*b* = 1.35, *SE* = .238, *t(67)* = 5.680, *p <* .001).

#### Analyses Model 4.3 – Social Support – Friends and Parenting Stress, Mediated by Paternal Depressive Symptoms

The indirect effect of social support – friends on PS through paternal depressive symptoms was significant; *b* = −4.382, *SE* = 1.446, 95% CI [−7.323, −1.661], (*n* = 70). The conditional direct effect of social support – friends on PS (c’ path) was non-significant *b* = −.335, *SE* = 1.733, *t(67)* = −.193, *p* = .847. The pathway between social support – friends and paternal depressive symptoms (a path) was significant (*b* = −2.981, *SE* = .808, *t(68)* = −3.689, *p <* .001), and the pathway between paternal depressive symptoms and PS (b path) was significant (*b* = 1.470, *SE* = .237, *t(67)* = 6.192, *p <* .001).

#### Analyses Model 4.4 – Social Support – Significant Other and Parenting Stress, Mediated by Paternal Depressive Symptoms

The indirect effect of social support – significant other on PS through paternal depressive symptoms was significant; *b* = −4.955, *SE* = 1.630, 95% CI [−8.488, −2.155], (*n* = 70). The conditional direct effect of social support – significant other on PS (c’ path) was non-significant *b* = −2.284, *SE* = 1.971, *t(67)* = −1.159, *p* = .251. The pathway between social support – significant other and paternal depressive symptoms (a path) was significant (*b* = −3.625, *SE* = .899, *t(68)* = −4.033, *p <* .001), and the pathway between paternal depressive symptoms and PS (b path) was significant (*b* = 1.367, *SE* = .239, *t(67)* = 5.722, *p <* .001).

## Discussion

Our study sought to determine the mediating role of paternal depression in the relationship between social support and PS. As predicted, social support from any source was negatively correlated with paternal depressive symptoms and PS. These findings align with the literature (Kim & Swain, 2007; Song et al., 2021). Paternal depressive symptoms were positively correlated with PS, consistent with our prediction and previous literature (Yim et al., 2015). Further, fathers reported significantly more social support from significant others than family or friends. This is consistent with previous literature that suggests men seek most of their support from their partners (Van Daalen et al., 2005). That is, fathers’ partners play a crucial role in men’s well-being by providing a space to feel understood and supported (Rominov et al., 2018).

Our study aimed to explore the association between social support from any source and PS through paternal depressive symptoms. The results indicated that total social support and support from each source indirectly affect PS through paternal depressive symptoms. This suggests that social support from any source reduces paternal depressive symptoms which in turn reduce PS. This confirms our hypothesis based on the direct relationships between social support and PS (Song et al., 2021), social support and depressive symptoms (Castle et al., 2008), and depressive symptoms and PS (Yim et al., 2015). Fathers are often recognized as support providers for their partners (Kim & Swain, 2007). They are expected to provide support to their partners and children, rather than request support for themselves, and they may question whether their personal needs are valid (Darwin et al., 2017). Considering the stigma fathers face around seeking support, it is understandable that many hesitate to ask for social support (Darwin et al., 2017). Lack of social support from friends, family, or significant others leaves fathers without the necessary emotional or tangible support to protect themselves against depressive symptomology (Kim & Swain, 2007). Consistent with the literature, our study found that as depressive symptoms increase, PS worsens (Yim et al., 2015). Negative attentional bias and low self-esteem are among potential depressive symptoms that may make fathers more susceptible to PS (Deater-Deckard, 2004). These symptoms may result in fathers perceiving greater PS, having worse psychological resilience, and believing they have poor parenting efficacy and are unable to cope with PS (Crnic & Ross, 2017; Deater-Deckard, 2004; O’Neil et al., 2009). Conversely, when fathers perceive accessible social support, their risk of depressive symptoms decreases, allowing them to better cope with PS.

### Limitations

The results of our study should be considered within the context of some limitations. First, the generalizability of the sample may be limited due to potential self-selection bias. The type of father who opted to participate in this study likely differed from those who did not, given that the findings speak to the experiences of fathers who experience high levels of social support and depressive symptoms compared to reports among similar samples (Fierloos et al., 2022; Vilagut et al., 2016). Considering that men are a difficult population to recruit for research (Yaremych & Persky, 2022), the type of men who volunteered to participate may have been more likely to discuss sensitive questions about their mental health than typical populations of men. Most of the study population was recruited via social media (86.3%) and belonged to at least one type of organized parenting social support (56.8%). This recruitment method may have captured fathers who are heavily involved in child-rearing and are motivated to share their parenting experiences. Further, fathers in this study were predominantly White, married, and had high socioeconomic status, despite extensive efforts to recruit a diverse sample of fathers using recommended recruitment methods for fathers (Yaremych & Persky, 2022). Given the factors studied in this article are culturally bound (i.e., social support; Zhai & Du, 2022), it is important for future research to consider the present research questions in additional groups.

Another element limiting our study was the use of questionnaires. Self-report questionnaires risk social desirability influencing participants’ responses, which can result in over- or under-reporting experiences (Latkin et al., 2017). This was a particular concern within the context of our study given that fathers may face unrealistic expectations and stigma for issues related to parenting and mental health (Darwin et al., 2017). While there is growing literature on the use of male-sensitive measures of depression symptoms (Rice et al., 2019), using the CES-D ensured findings are comparable to the mothering literature.

### Implications

Based on the indirect effect of social support on PS through paternal depressive symptoms, there is a need for social support from family, friends, and significant others to improve fathers’ depressive symptomology and PS. Fathers should prioritize social support and attend groups that provide opportunities to build social connection. This call for support is urgent given that some men do not recognize the need for social support (Skarsater et al., 2003), fathers face unique parenting experiences (Chalmers & Meyer, 1996), low social support (Levy & Kotelchuck, 2022), and feel more unprepared for parenthood compared to mothers (Darwin et al., 2017). Fathers also may seek support for their well-being reactively, once they need to resolve a specific problem, and tend to not engage in mental health maintenance (Rominov et al., 2018). This suggests there is a need for societal changes around prioritizing social connections between men. Our findings urge men to form and join group activities (i.e., sports, book clubs, game nights, parenting groups etc.) to build new and strengthen existing relationships. Men should be educated on the importance of maintaining these groups from a young age.

Existing stigma around men seeking social support may contribute to men’s social isolation (Wester et al., 2007). Men may avoid social and emotional connectedness in an attempt to maintain a traditional masculine sense-of-self, which can develop as a result of harsh male socialization (Blazina et al., 2007). Men may strive to be ‘independent’ and dismiss their need for emotional support (McKenzie et al., 2018). They may resort to humor to cope with emotional conversations and may rely on relationships with women for emotional support over relationships with other men (McKenzie et al., 2018). Some fathers are hesitant to discuss mental health concerns with friends due to stigma (Rominov et al., 2018). While women engage in more communication about intimate topics, men communicate more to exchange instrumental information (Psylla et al., 2017). Moreover, fathers question whether their PS is valid (Darwin et al., 2017) and are viewed as support-givers for their partners (Kim & Swain, 2007). Societal changes are required to address these perceptions to encourage fathers to engage their friends, family, and partners in conversations around their personal needs and well-being. Informing fathers of the importance of getting support for themselves to increase their whole family’s well-being, could reduce stigma and motivate fathers to seek more support (Rominov et al., 2018). Further, the family, friends, and partners of fathers are urged to be more receptive and encouraging of these conversations. Public education initiatives may be used to teach the public how to support fathers, offer help, and listen to and validate their feelings.

Overall, the takeaway for fathers is that taking care of their own mental health and seeking support from friends, family, and partners will have benefits for the family unit. This study supports the need for fathers to seek social support proactively, not just when challenges become unbearable. Clinicians working with fathers who have difficulty managing parenting responsibilities should be aware of the possibility of co-occurring problems with depressive symptoms and limited social support. Clinicians should also note the importance of engaging fathers in family therapy to address their concerns to improve family functioning.

## Conclusion

The present study highlights the importance of social support and paternal depressive symptoms on PS. Findings elucidated that total social support and social support from friends, family, and a significant other each exert an effect on PS through paternal depressive symptoms. These findings contribute to knowledge of the complex interplay between social support, paternal depressive symptoms and PS. It emphasizes the need for research, clinical and societal changes to support fathers’ mental health and family functioning. Ultimately, the current findings contribute to the growing body of literature on fathers’ mental health and provide a foundation for future investigations.

## Supplemental Material

Supplemental Material - The Impact of Social Support: Fathers’ Depressive Symptoms and Parenting Stress Supplemental Material for The Impact of Social Support: Fathers’ Depressive Symptoms and Parenting Stress by Emily Hogan, Dana Ronaghan, Karis Cochrane, Alyssa Romaniuk, Lara Penner-Geoke, Taryn Gaulke, and Jen Theule in Journal of Family Issues
