# Supplementary material for: The Impact of Social Support: Fathers’ Depressive Symptoms and Parenting Stress
Source: J Fam Issues. 2025 Mar 18;46(6):1028–49. doi: 10.1177/0192513X251322143 (PMC12013979; doi:10.1177/0192513X251322143)
Supplement: Supplemental Material - The Impact of Social Support: Fathers’ Depressive Symptoms and Parenting Stress [file sj-pdf-1-jfi-10.1177_0192513X251322143.pdf]

Supplemental Material

Figure 1

Mediation Model 4.1

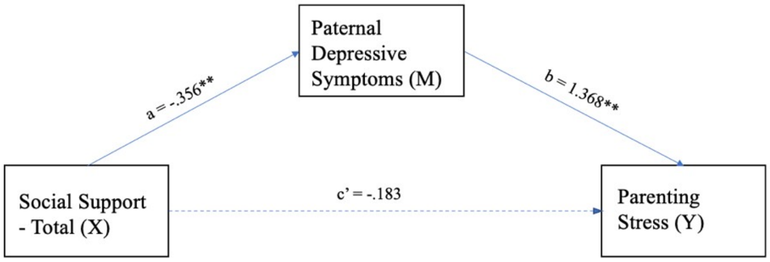

Table 4

Mediation Model 4.1 Results

	Consequent											
Antecedent	Paternal Depressive Symptoms (M)						Parenting Stress (Y)					
		Coeff.	*SE*	*p*	LCI	UCI		Coeff.	*SE*	*p*	LCI	UCI
Social Support – Total (X)	*a*	-.356	.078	.000	-.512	-.200	*c'*	-.183	.181	.316	-.544	.178
Paternal Depressive Symptoms (M)		-	-	-	-	-	*b*	1.368	.246	.000	.878	1.859
Constant	i_M	37.213	5.095	.000	27.046	47.380	i_Y	78.675	13.795	.000	51.140	106.210
	$R^2 = .234$<br>$F(1,68) = 20.789, p < .001$						$R^2 = .422$<br>$F(2,67) = 24.454, p < .001$					

Note. In this model M is the mediator variable, X is the independent variable, Y is the dependent variable, i_M and i_Y are the intercepts for each regression line, and *a*, *b*, and *c'* are regression coefficients.

Table 5

Mediation Model 4.2 Results

	Consequent											
Antecedent	Paternal Depressive Symptoms (M)						Parenting Stress (Y)					
		Coeff.	*SE*	*p*	LCI	UCI		Coeff.	*SE*	*p*	LCI	UCI
Social Support – Family (X)	*a*	-3.448	.862	.000	-5.167	-1.729	*c'*	-2.491	1.878	.189	-6.240	1.257
Paternal Depressive Symptoms (M)		-	-	-	-	-	*b*	1.350	.238	.000	.876	1.825
Constant	i_M	32.822	4.708	.000	23.427	42.217	i_Y	80.497	12.090	.000	56.365	104.629
	$R^2 = .191$<br>$F(1,68) = 16.020, p < .001$						$R^2 = .428$<br>$F(2,67) = 25.084, p < .001$					

Table 6

Mediation Model 4.3 Results

	Consequent											
Antecedent	Paternal Depressive Symptoms (M)						Parenting Stress (Y)					
		Coeff.	*SE*	*p*	LCI	UCI		Coeff.	*SE*	*p*	LCI	UCI
Social Support – Friends (X)	*a*	-2.981	.808	.000	-4.594	-1.369	*c'*	-.335	1.733	.847	-3.794	3.124
Paternal Depressive Symptoms (M)		-	-	-	-	-	*b*	1.470	.237	.000	.996	1.944
Constant	i_M	29.745	4.282	.000	21.200	38.289	i_Y	67.282	10.959	.000	45.409	89.155
	$R^2 = .167$<br>$F(1,68) = 13.608, p < .001$						$R^2 = .414$<br>$F(2,67) = 23.616, p < .001$					

Table 7

Mediation Model 4.4 Results

	Consequent											
Antecedent	Paternal Depressive Symptoms (M)						Parenting Stress (Y)					
		Coeff.	*SE*	*p*	LCI	UCI		Coeff.	*SE*	*p*	LCI	UCI
Social Support – Significant Other (X)	*a*	-3.625	.899	.000	-5.419	-1.831	*c'*	-2.284	1.971	.251	-6.219	1.650
Paternal Depressive Symptoms (M)		-	-	-	-	-	*b*	1.367	.239	.000	.890	1.844
Constant	i_M	34.587	5.099	.000	24.411	44.763	i_Y	79.689	13.006	.000	53.728	105.650
	$R^2 = .193$<br>$F(1,68) = 16.263, p < .001$						$R^2 = .425$<br>$F(2,67) = 24.729, p < .001$					
